# Functional connectivity as a prognostic biomarker for neurodevelopmental outcomes in preterm infants without severe brain injury

**DOI:** 10.1093/braincomms/fcaf476

**Published:** 2025-12-16

**Authors:** Yi-Tien Li, Li-Wen Chen, Chia-Lin Koh, Yung-Chieh Lin, Chao-Ching Huang

**Affiliations:** Translational Imaging Research Center, Taipei Medical University Hospital, Taipei 11031, Taiwan; Research Center for Neuroscience, Taipei Medical University, Taipei 11031, Taiwan; Ph.D. Program in Medical Neuroscience, College of Medical Science and Technology, Taipei Medical University, Taipei 11031, Taiwan; Department of Pediatrics, National Cheng Kung University Hospital, College of Medicine, National Cheng Kung University, Tainan 70100, Taiwan; Department of Occupational Therapy, College of Medicine, National Cheng Kung University, Tainan 70100, Taiwan; Department of Pediatrics, National Cheng Kung University Hospital, College of Medicine, National Cheng Kung University, Tainan 70100, Taiwan; Department of Pediatrics, Shuang Ho Hospital, Taipei Medical University, New Taipei 23561, Taiwan; Department of Pediatrics, College of Medicine, Taipei Medical University, Taipei 11031, Taiwan

**Keywords:** preterm infants, neurodevelopmental impairment, functional connectivity, thalamo-cortical circuit, thalamo-subcortical circuit

## Abstract

Despite a decline in severe neonatal brain injury in preterm infants, neurodevelopmental impairment remains prevalent. Identifying early biomarkers for neurodevelopmental impairment, particularly in infants without severe neonatal brain injury, is crucial for intervention. This study explores whether brain dysmaturation, indicated by functional connectivity alterations at term-equivalent age, predicts neurodevelopmental impairment severity at 24 months corrected age in preterm infants without severe neonatal brain injury. In this observational cohort study, preterm infants born < 31 weeks’ gestation without severe neonatal brain injury underwent resting-state functional MRI at term-equivalent age. Neurodevelopmental outcomes at corrected age 24 months were assessed using Bayley-III cognitive and motor composite scores, cerebral palsy severity, and neurosensory impairments. Functional connectivity alterations were analyzed in relation to cognitive, language, and motor outcomes. Machine learning models were applied to assess the predictive value of functional connectivity features alongside neonatal exposures for neurodevelopmental impairment severity. Among the 122 preterm infants, 89 (73%) infants had no/mild neurodevelopmental impairment, 27 (22%) had moderate neurodevelopmental impairment, and 6 (5%) showed severe neurodevelopmental impairment. Compared with the no/mild neurodevelopmental impairment group, the moderate/severe neurodevelopmental impairment group was significantly lower in gestational age, and required longer durations of invasive mechanical ventilation, oxygen therapy, vasopressors, and total parenteral nutrition during admission. Compared with term-born controls, a clear trend emerged across neurodevelopmental impairment severity levels: as impairment increased from the no/mild group to the moderate and severe groups, the clustering coefficient increased, whereas the global efficiency decreased. Statistical comparisons between the no/mild and moderate/severe groups, relative to term-born controls, confirmed these patterns (clustering coefficient: *t* = −4.38, *P* < 0.001; global efficiency: *t* = 3.44, *P* < 0.001). Infants with no/mild neurodevelopmental impairment exhibited enhanced connectivity in the limbic system (*t* = −5.21, *P* < 0.001) and between the thalamus and basal ganglia (*t* = −5.9, *P* < 0.001), but this compensatory connectivity weakened with increasing neurodevelopmental impairment severity. The thalamo-cortical (frontal lobe, limbic system), thalamo-basal ganglia, and thalamo-cerebellar connectivity were strongly associated with cognitive, language, and motor performance at follow-up. A predictive model incorporating these functional connectivity features and neonatal adverse exposure parameters achieved 82% accuracy. Distinct disruptions in functional connectivity at term-equivalent age in very preterm infants without severe neonatal brain injury may predict the severity of later neurodevelopmental impairment. Early functional connectivity assessment holds promise as a biomarker for identifying high-risk infants who may benefit from timely neurodevelopmental interventions.

## Teaser

Li *et al.* report that functional connections between the thalamus and cortical and subcortical regions at term-equivalent age predict severity of neurodevelopmental impairment outcomes at two years in preterm infants, highlighting early brain connectivity as a potential marker for later neurodevelopment.

## Graphical Abstract

**Graphical Abstract:**
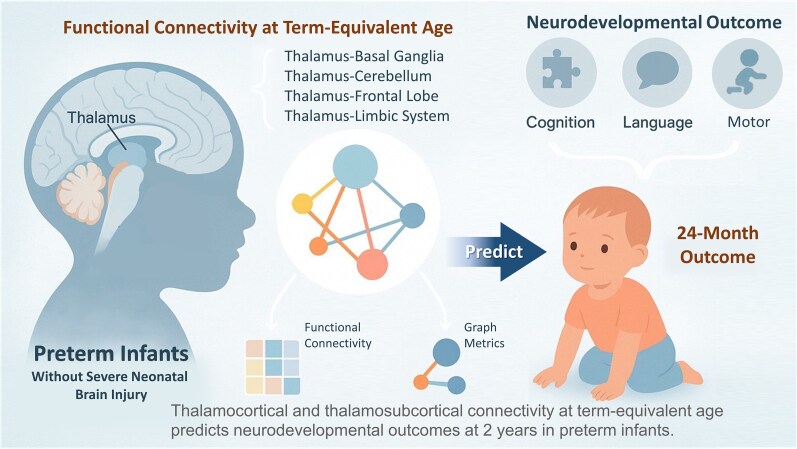

## Introduction

Improving perinatal health and neonatal critical care over the past decades have substantially increased the survival of infants born with gestational age (GA) < 32 weeks.^1,2^ These very preterm infants are at risks of severe neonatal brain injury, such as high-grade intraventricular hemorrhage (IVH), cystic periventricular leukomalacia, or cerebellar hemorrhage.^3^ These types of severe neonatal brain injury are the most important determinants of their neurodevelopmental impairment (NDI) outcomes at follow-up.^3-6^ Despite the declining rates of mortality and severe neonatal brain injury in recent years,^2,7,8^ the incidence of NDI still remain high at an average of 30–40%, without significant improvement among preterm survivors.^1,7,9^ In this era, focus has shifted from preventing mortality to reducing NDI outcomes,^3^ especially in infants without severe neonatal brain injury.

Very preterm infants are born with rather smooth immature cortex that is supposed to be developed into the complex system of gyrus/sulci and connectivity in the third trimester of gestation during pregnancy.^10^ In this extra-uterine period, very preterm infants need to build the brain circuits that underlie their future development of cognitive, speech, and motor performance,^11^ while under adverse exposures in the neonatal intensive care unit (NICU).^12,13^ The extra-uterine progress of brain functional connectivity (FC) development depicted by magnetic resonance imaging (MRI) may be disrupted under diverse adverse exposures in the NICU.^12,14-16^ The aberrant brain connectivity noted at term-equivalent age is likely associated with NDI outcomes at follow-up.^3,12,17^ In this era of declining severe neonatal brain injury, however, very few studies have focused on the relationship between FC patterns by resting state fMRI at term-equivalent age and different severity of NDI outcomes at follow-up among infants without severe neonatal brain injury.

Prior studies have demonstrated that alterations in brain connectivity at term-equivalent age are associated with later cognitive and behavioural outcomes in preterm populations. For instance, FC patterns in early life have been linked to domain-specific developmental trajectories,^18^ while structural thalamocortical integrity has been shown to associate with cognitive performance at age two.^19^ Moreover, longitudinal neuroimaging has identified associations between early brain development and environmental or socio-demographic risk factors.^20^ While previous studies have demonstrated that alterations in brain FC at term-equivalent age are associated with later neurodevelopmental outcomes in preterm infants, most have focused on specific domains—such as cognitive or language scores^18,21^ —or have aggregated these into composite psychomotor outcomes using instruments like the Bayley Scales of Infant and Toddler Development (BSID-III).^20^ However, these domain-specific approaches may not fully capture the broader clinical burden of NDI outcomes, which also includes motor and cognitive dysfunction, cerebral palsy (CP), and neurosensory deficits. Furthermore, few studies have stratified outcomes by severity of NDI outcomes or examined whether network-level FC dysmaturation by term-equivalent age can serve as a robust early biomarker to predict the graded levels of impairment outcomes, particularly among infants without severe neonatal brain injury. This gap limits the development of early risk stratification tools that integrate brain-based biomarkers with clinical risk exposures to guide timely intervention in the most vulnerable preterm infants.

In very preterm infants without severe neonatal brain injury, it remains unclear whether infants with different severity of NDI outcomes (no/mild, moderate or severe NDI) at follow-up already exhibit distinct alterations in the FC at term-equivalent age. In addition, given the functional importance of hubs, it is important to understand how the patterns of FC at term-equivalent age shape neurodevelopmental outcomes, such as cognition, language, and motor performance. Comprehensive examinations of different dysmaturational patterns of FC may help facilitate to develop a prediction model for NDI outcomes.

## Materials and methods

### Participants

This prospective cohort study was conducted at the National Cheng Kung University Hospital from April 2019 to February 2022 and was approved by the Institutional Review Board of National Cheng Kung University Hospital (A-BR-108-013). The study adhered to the principles of the Declaration of Helsinki. Written informed consent for clinical data collection, MRI examinations, and neurodevelopmental follow-up was obtained from the parents during hospitalization.

**Figure 1:**
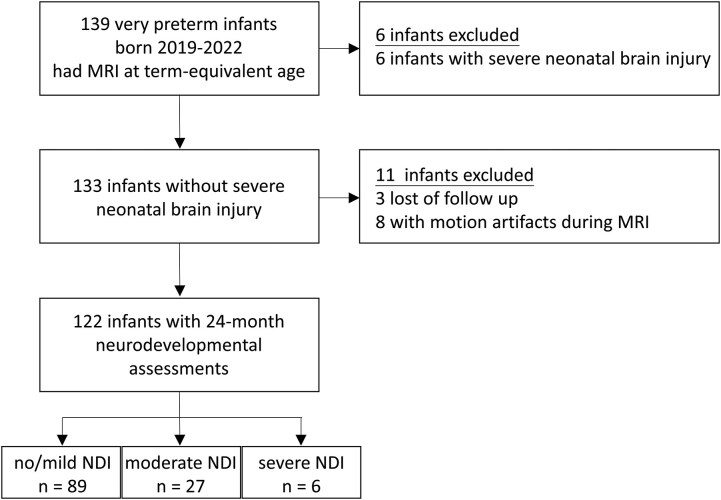
**Flow diagram.** A schematic representation of the 139 very preterm-birth infants whose parents gave consent for MRI examination by term equivalent age, detailing the inclusion and exclusion criteria and the final grouping of infants based on the severity of NDI outcomes at 24 months corrected age. Group sizes: term-born controls (*N* = 40), no/mild NDI (*N* = 89), moderate NDI (*N* = 27), severe NDI (*N* = 6). NDI, neurodevelopmental impairment.

The study focused on infants born before 31 weeks of gestation who survived to undergo MRI examinations at term-equivalent age, and neurodevelopmental assessment at 24 months corrected age (*N* = 139; 80 males, 59 females; Fig. 1). The exclusion criteria included infants with severe neonatal brain injury (grade III IVH, IVH with periventricular hemorrhage, cystic periventricular leukomalacia, or cerebellar hemorrhage) confirmed by serial cranial ultrasonography or MRI at term-equivalent age, congenital or chromosomal abnormalities, or significant motion artifacts during MRI examinations. To minimize motion-related confounds, infants with >10% of fMRI frames showing framewise displacement >0.5 mm were excluded in addition to visual inspection.

### Demographics and neonatal risk exposures

Demographics included maternal educational levels and family socioeconomic status. Perinatal complications, including preeclampsia, small for gestational age (SGA), and neonatal adverse exposures during admission were obtained from the electronic medical system.^22-24^ Neonatal adverse exposures included durations of requiring invasive mechanical ventilation (IMV), supplemental oxygen, vasopressor for altered hemodynamics and total parenteral nutrition, hemodynamically significant patent ductus arteriosus requiring surgical ligation, use of postnatal steroid, the postnatal days reaching full feeding, sepsis, and necrotizing enterocolitis (NEC).^25^

### Severity of NDI outcomes

Neurodevelopmental outcomes at a corrected age of 24 months were performed using the Bayley Scales of Infant Development, third edition (BSID-III) by a child psychologist who was blind to the infants’ past histories. The cognitive, language, and motor composite scores of the BSID-III have a mean of 100 and a standard deviation (SD) of 15 points, with scores > 85 as no/mildly low score, between 85 and 70 as moderately low score, and < 70 as severely low score.^25^ The scaled scores of the BSID-III, including cognition, receptive communication (RC), expressive communication (EC), fine motor (FM), and gross motor (GM) functions are standardized to a mean of 10 and a SD of 3.^25^ CP was diagnosed by child neurologists on the basis of abnormal muscle tonicity and activity limitations by Gross Motor Function Classification System (GMFCS).^25^ The severity of CP was determined using the GMFCS levels and classified as mild (level 1), moderate (levels 2-3), and severe (levels 4-5).

According to the criteria used in Bell *et al*.’s study,^2^ the NDI outcomes were categorized into no/mild (Bayley-III cognitive and motor composite score > −1 SD or higher, and GMFCS level 0 or 1), moderate (Bayley-III cognitive or motor composite score between −1 and −2 SD, or GMFCS level 2 or 3), and severe (Bayley-III cognitive or motor composite score < −2 SD, GMFCS level 4 or 5, bilateral blindness or deafness).^2,25^

### MRI data acquisition

MRI scans were performed in preterm infants using a Philips 3T Ingenia scanner (Philips Healthcare, Netherlands) equipped with an 8-channel dStream (dS) Pediatric NeuroSpine coil. Imaging was conducted during natural sleep or under mild sedation with chloral hydrate (50 mg/kg), administered according to standard clinical guidelines to minimize motion artifacts.^26^ The imaging protocol included high-resolution T1-weighted images, T2-weighted images, and resting-state functional MRI (rsfMRI). T1-weighted images were acquired using a fast spin echo sequence with a repetition time (TR) of 9.4 ms, echo time (TE) of 4.5 ms, flip angle of 7°, voxel size of 1 × 1 × 1 mm³, and a matrix size of 192 × 192 × 128. T2-weighted images were obtained with a TR of 3000 ms, TE of 19.7 ms, flip angle of 90°, voxel size of 1 × 1 × 1 mm³, and a matrix size of 192 × 192 × 128. For rsfMRI, a single-shot gradient-echo echo-planar imaging sequence was used with a TR of 2000ms, TE of 35 ms, flip angle of 90°, voxel size of 3 × 3 × 4 mm³, and 180 volumes. The total scan time for acquiring 180 volumes at a TR of 2000ms was ∼6 min.

Preprocessing of rsfMRI data was conducted using Statistical Parametric Mapping software (SPM12; Wellcome Department of Imaging Neuroscience, University College London, UK). The initial ten frames were discarded to allow for magnetization equilibrium, and the remaining images underwent slice timing correction and realignment to the first volume to correct for head motion. Framewise displacement was computed for each subject, and subjects with >10% of volumes exceeding framewise displacement >0.5 mm were excluded from further analysis. Co-registration with the corresponding T2-weighted images was performed, followed by normalization to the Developing Human Connectome Project (dHCP) infant brain template.^27,28^ To enhance the signal-to-noise ratio, spatial smoothing with a 3-mm isotropic Gaussian kernel was applied. Temporal filtering using a bandpass range of 0.01–0.08 Hz was conducted to remove low-frequency drift and high-frequency noise. Nuisance regressors, including six motion parameters, average white matter signal, and cerebrospinal fluid signal, were incorporated to reduce confounding noise.

MRI data from another 40 term-born neonates (GA = 38.99 ± 1.69 weeks) were obtained from the dHCP dataset ^29^ and underwent the same preprocessing pipeline as the preterm cohort. These infants were selected based on the absence of major brain injury, normal birth history, and availability of complete imaging datasets. The sex distribution and postmenstrual age at MRI in the term-born group did not differ significantly from any of the three preterm subgroups (no/mild, moderate, and severe NDI), supporting their suitability as a reference group for FC comparisons at term-equivalent age. To minimize potential scanner-related bias, the term-born dHCP rsfMRI data were temporally resampled to match the repetition time (TR = 2000ms) and acquisition duration (360 s) of our institutional preterm cohort. All rsfMRI data underwent an identical preprocessing pipeline based on the dHCP neonatal preprocessing workflow,^30,31^ (including slice-timing correction, motion realignment, spatial normalization to a standard neonatal template, band-pass temporal filtering (0.01–0.08 Hz), and nuisance regression (six motion parameters, white matter, and CSF signals). Given that graph theoretical metrics such as clustering coefficient and global efficiency are relatively robust to site-related confounds,^32,33^ and that term-born controls were included only for descriptive reference, no further harmonization was performed.

### Functional connectivity and graph theory analysis

Resting-state FC was computed by extracting the mean time series from predefined regions of interest (ROIs) based on the dHCP neonatal brain atlas.^27,28^ Each subject's brain was parcellated into 87 ROIs spanning cortical and subcortical structures. From the full atlas, 49 ROIs were selected (Supplementary Table 2), encompassing both cortical and subcortical grey matter regions. White matter areas and non-parenchymal structures such as cerebrospinal fluid and ventricles were excluded to ensure accurate representation of neuronal functional activity. Pearson correlation coefficients were calculated between each pair of the 49 ROIs, resulting in a 49 × 49 symmetric correlation matrix for each participant. These coefficients were Fisher Z-transformed to enable statistical comparison. Graph theory analysis was employed to evaluate the topological properties of whole-brain functional networks.^34,35^ The correlation matrices were thresholded to generate binarized adjacency matrices at a fixed network density (30%), ensuring consistent network sparsity across participants. Two key metrics were computed:

- Clustering coefficient (unitless scalar) quantifies the tendency of a node’s neighbors to also be connected to each other, reflecting local segregation within the network.
- Global efficiency (unitless scalar) measures the inverse of the average shortest path length between all pairs of nodes, representing global integration of information flow across the brain.

Both metrics were computed from the binarized network using the in-house MATLAB (version R2020a; Mathworks, Sherborn, MA, USA) scripts, and averaged across all nodes to yield a single scalar value per metric for each subject.

### Outcome measures and statistical analysis

The primary outcome was to evaluate association between FC metrics at term-equivalent age and NDI severity outcomes. To compare FC metrics and neonatal adverse exposures between NDI severity groups, two-tailed two-sample *t*-tests were performed. The relationships between FC patterns and the composite scores for cognitive, language, and motor outcomes were analyzed using partial correlation analyses, controlling for potential confounding variables, including the postmenstrual age at MRI examinations and sex. Additional partial correlation analyses were performed between FC metrics and individual Bayley-III subscale scores, including RC, EC, FM, and GM domains, to further explore domain-specific associations. All correlations were computed across the entire preterm cohort rather than within individual outcome subgroups. False discovery rate (FDR) correction was applied separately within each family of hypotheses, including (i) the three Bayley-III composite scores (cognitive, language, and motor), (ii) the five Bayley-III subscale scores (RC, EC, FM, GM, and cognition), and (iii) each graph-theoretical metric (clustering coefficient and global efficiency). A significance threshold of *q* < 0.05 was applied to account for multiple comparisons, ensuring robust statistical interpretation.

To evaluate the predictive value of FC metrics, we first applied Least Absolute Shrinkage and Selection Operator (Lasso) regression for feature selection and regularization to reduce overfitting risk.^36^ We then tested multiple supervised machine learning algorithms, including decision tree, support vector machine (SVM), naïve Bayes, K-nearest neighbors (KNN), discriminant analysis, and ensemble classifiers. The primary classification task was to predict categorical NDI severity groups, specifically distinguishing between no/mild group and moderate/severe group, based on FC and clinical variables. Given the unequal class sizes (no/mild: *N* = 89; moderate: *N* = 27; severe: *N* = 6), class weights proportional to the inverse of class frequencies were applied during model training to mitigate the impact of imbalance among NDI severity groups, a widely used approach to reduce classifier bias in imbalanced datasets.^37,38^ Grid search was used to tune hyperparameters, and model performance was assessed via leave-one-out cross-validation (LOOCV).^39^ Classification accuracy and area under the receiver operating characteristic curve (ROC-AUC) were used as the primary performance metrics. All analyses were conducted using MATLAB (R2020a; MathWorks, Sherborn, MA, USA).^36,39^

## Results

### Patient characteristics

Among the 139 very preterm infants who survived to receive MRI examinations at term-equivalent age after obtaining consents from their parents, 6 infants had severe neonatal brain injury. Of the 133 infants without severe neonatal brain injury, 130 (98%) infants had received neurodevelopmental assessment at corrected age 24 months. After excluding 8 infants with marked motion artifacts during examinations, either by visual inspection or based on exceeding the framewise displacement threshold (>10% of frames with framewise displacement >0.5 mm), neuroimages were available for analysis in 122 infants. Of the 122 infants, 89 (73%) infants had no/mild NDI, 27 (22%) had moderate NDI, and 6 (5%) had severe NDI (Fig. 1 and Table 1).

**Table 1:** Differences in demographics, neonatal exposures, and neurodevelopmental performance at corrected age 24 months among three different NDI outcome groups in preterm infants without severe neonatal brain injury

Variable	No/Mild NDI *N* = 89	Moderate NDI *N* = 27	Severe NDI *N* = 6	*P* value ^1^	Moderate/Severe NDI *N* = 33	*P* value ^2^
Family social risks						
Mother education, below university, *n* (%)	33 (37)	15 (56)	3 (50)	0.214	18 (55)	0.082
Low family socioeconomic status, *n* (%)	14 (16)	4 (15)	2 (33)	0.513	6 (18)	0.745
Risks at perinatal period and birth						
Preeclampsia, *n* (%)	17 (19)	3 (11)	0 (0)	0.333	3 (9)	0.185
Clinical chorioamnionitis, *n* (%)	19 (21)	7 (26)	3 (50)	0.268	10 (30)	0.302
Gestational age, weeks, mean ± SD	27.4 ± 2.2	26.2 ± 2.7	26.0 ± 2.2	**0**.**047**	26.2 ± 2.6*	**0**.**015**
Body weight, *z* scores, mean ± SD	−0.13 ± 0.74	−0.29 ± 0.85	−0.31 ± 0.35	0.548	−0.3 ± 0.8	0.272
Head circumference, *z* scores, mean ± SD	−0.22 ± 0.90	−0.34 ± 1.05	−0.57 ± 1.11	0.815	−0.4 ± 1.0	0.536
Small for gestational age, *n* (%)	4 (5)	4 (15)	0 (0)	0.132	4 (12)	0.131
Male, *n* (%)	49 (55)	20 (74)	4 (67)	0.198	24 (73)	0.077
5-min Apgar score <7, *n* (%)	19 (21)	10 (37)	1 (17)	0.227	11 (33)	0.172
Exposures during admission						
Duration of IMV, days, mean ± SD	5.6 ± 10.1	12.7 ± 16.2	9.0 ± 12.8	0.073	12.1 ± 15.5*	**0**.**023**
Duration of oxygen therapy, days, mean ± SD	21.2 ± 21.4	30.7 ± 21.4	27.7 ± 17.0	0.068	30.2 ± 20.5*	**0**.**021**
hs-PDA requiring ligation, *n* (%)	14 (16)	6 (22)	1 (17)	0.736	7 (21)	0.476
Duration of vasopressor use, days, mean ± SD	2.8 ± 4.4	6.1 ± 7.9	3.7 ± 5.5	0.060	5.6 ± 7.5*	**0**.**029**
Duration of total parental nutrition, days, mean ± SD	23.6 ± 12.9	31.0 ± 16.2	29.5 ± 17.2	0.126	30.7 ± 16.1*	**0**.**042**
Grade I/II IVH, *n* (%)	28 (32)	12 (44)	3 (50)	0.344	15 (46)	0.151
Sepsis, *n* (%)	16 (18)	7 (26)	1 (17)	0.649	8 (24)	0.439
Necrotizing enterocolitis, *n* (%)	4 (5) ^a^	1 (4) ^ab^	2 (33) ^b^	**0**.**012**	3 (9)	0.332
Use of postnatal steroid, *n* (%)	6 (7)	4 (15)	0 (0)	0.308	4 (12)	0.457

hs-PDA, hemodynamically significant patent ductus arteriosus; IMV, invasive mechanical ventilation; IVH, intraventricular hemorrhage; NEC, necrotizing enterocolitis; necrotizing enterocolitis: with stage ≥ IIa. *P* value¹ was calculated using one-way ANOVA or the Kruskal–Wallis test for continuous variables, and the chi-squared test for categorical variables. *Post hoc* comparisons among the No/Mild NDI, Moderate NDI, and Severe NDI groups were performed using Tukey’s HSD or Dunn’s test, as appropriate. Superscript letters (a, b) indicate statistically significant differences between groups.

*P* value² represents the comparison between the No/Mild NDI group and the combined Moderate/Severe NDI group, using independent *t*-tests or Mann–Whitney U-tests for continuous variables and chi-squared tests for categorical variables. An asterisk (*) indicates a statistically significant difference between the No/Mild NDI and Moderate/Severe NDI groups (*P* < 0.05). Statistically significant *P*-values (*P* < 0.05) are presented in bold.

The moderate or severe NDI group had significantly higher proportions of CP and hearing impairment than the no/mild NDI group. The no/mild NDI group also had significantly higher scores in cognition, language and motor performance than the moderate or severe NDI group (Table 2). The severe NDI group had a significantly higher proportion of CP and hearing impairment than the no/mild NDI or moderate NDI group.

**Table 2:** Neurodevelopmental outcomes at corrected age 24 months

	No/Mild NDI *N* = 89	Moderate NDI *N* = 27	Severe NDI *N* = 6	*P* value ^1^	Moderate/Severe NDI *N* = 33	*P* value ^2^
BSID-III Composite Scores						
Cognition, mean ± SD	100.2 ± 11.7^a^	82.8 ± 8.8^b^	85.0 ± 16.4^ab^	<0.001	83.2 ± 10.3*	<0.001
Language, mean ± SD	96.2 ± 12.5^a^	82.5 ± 8.7^b^	70.8 ± 10.0^b^	<0.001	80.4 ± 9.9*	<0.001
Motor, mean ± SD	96.6 ± 8.7^a^	82.6 ± 4.7^b^	73.5 ± 17.3^b^	<0.001	80.9 ± 8.8*	<0.001
BSID-III Scaled Scores						
Cognition, mean ± SD	10.0 ± 2.3^a^	6.6 ± 1.8^b^	7.0 ± 3.3^ab^	<0.001	6.6 ± 2.1*	<0.001
Receptive expression, mean ± SD	9.7 ± 2.3^a^	7.4 ± 1.5^b^	5.8 ± 2.6^b^	<0.001	7.2 ± 1.8*	<0.001
Expressive expression, mean ± SD	8.9 ± 2.3^a^	6.5 ± 2.0^b^	4.2 ± 1.3^b^	<0.001	6.1 ± 2.1*	<0.001
Fine motor, mean ± SD	9.9 ± 1.7^a^	7.0 ± 0.9^b^	6.3 ± 2.2^b^	<0.001	6.8 ± 1.2*	<0.001
Gross motor, mean ± SD	9.0 ± 1.8^a^	7.2 ± 1.1^b^	4.8 ± 3.9^b^	<0.001	6.8 ± 2.1*	<0.001
Hearing impairment, *n* (%)	0 (0)^a^	0 (0)^a^	2 (33)^b^	<0.001	2 (6)*	0.019
Cerebral Palsy, *n* (%)	0 (0)^a^	1 (4)^a^	3 (50)^b^	<0.001	4 (12)*	0.005

*P* value¹ was calculated using the Kruskal–Wallis test. *Post hoc* comparisons among the No/Mild NDI, Moderate NDI, and Severe NDI groups were performed using Dunn’s test. Superscript letters (a, b) indicate statistically significant differences between groups.

*P* value² represents the comparison between the No/Mild NDI group and the combined Moderate/Severe NDI group using Mann–Whitney U-test. An asterisk (*) indicates a statistically significant difference between the No/Mild NDI and Moderate/Severe NDI groups (*P* < 0.05).

The three NDI groups were comparable in maternal education, family socioeconomic status, and the proportions of clinical chorioamnionitis, preeclampsia and 5-min Apgar score < 7 in the perinatal period and at birth, and adverse exposures during admission except showing significant differences in GA and the rate of NEC (Table 1). Compared with the no/mild NDI group, the moderate or severe NDI group was significantly lower in GA, and required longer durations of IMV, oxygen therapy, vasopressors, and total parenteral nutrition during admission.

### Distinct FC patterns at term-equivalent age associated with the severity of NDI outcomes at age 24 months

**Figure 2:**
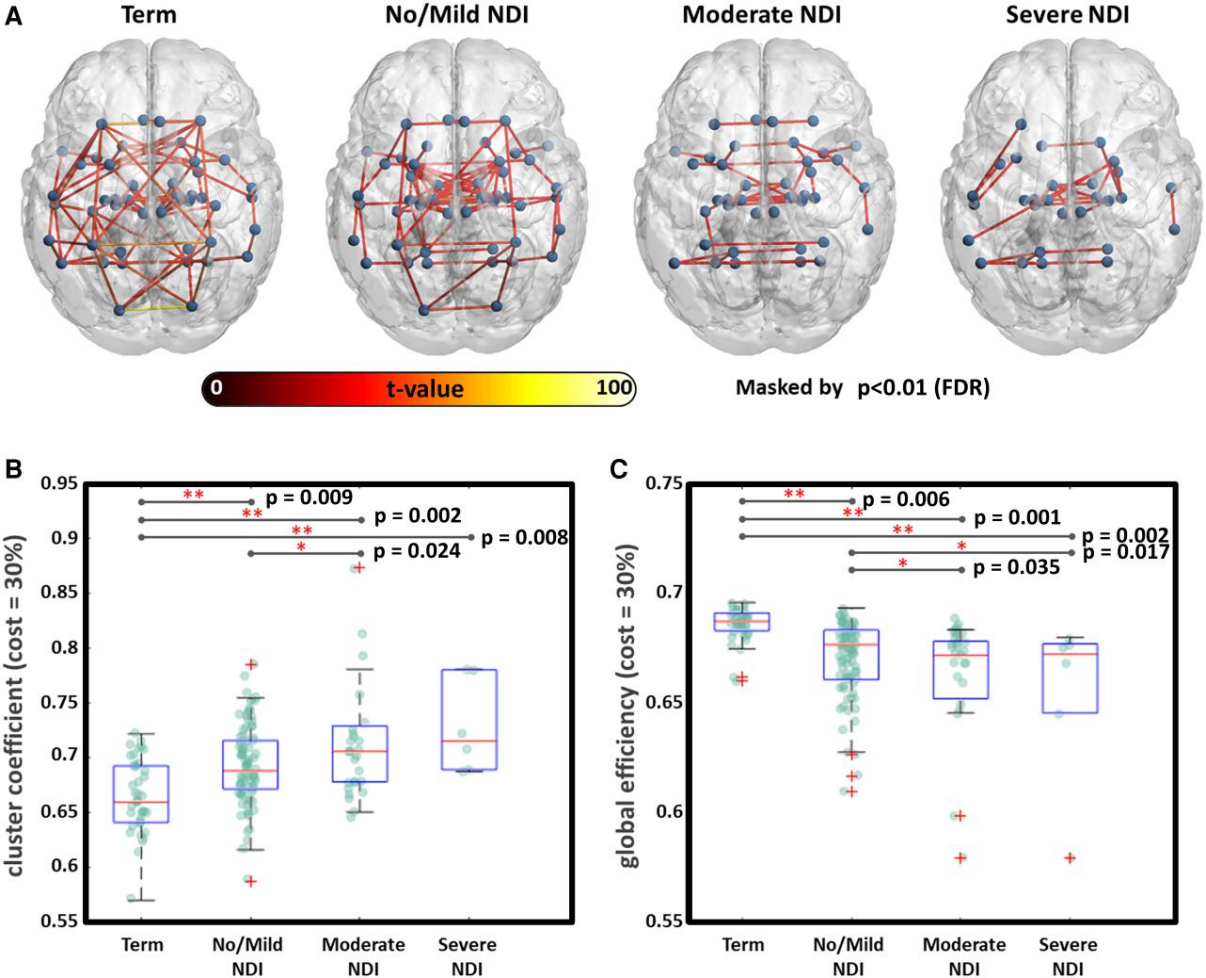
**Group differences in brain network topology at term-equivalent age.** (**A**) Overall differences in functional network organization between term-born controls (*N* = 40) and preterm infants with no/mild (*N* = 89), moderate (*N* = 27), and severe (*N* = 6) NDI outcomes at corrected age 24 months. (**B**) Clustering coefficient (reflecting local network connectivity), and (**C**) global efficiency (reflecting overall network integration) are displayed. Group comparisons were performed using two-sample *t*-tests with FDR correction. Exact *P*-values for all significant pairwise comparisons (e.g. term versus no/mild, term versus moderate, etc.) are displayed within the figure. The FC topologies were masked at a significance level of *P* < 0.01 with FDR correction, and graph theory metrics were calculated at a 30% cost threshold of the connectivity matrix. (Significance threshold: *P < 0.05 and **P < 0.01 with FDR correction). FDR, false discovery rate; NDI, neurodevelopmental impairment.

No significant differences were observed in postmenstrual age at MRI across the three different outcome groups (no/mild NDI, 41.4 ± 4.9 weeks; moderate NDI, 43.1 ± 4.7 weeks; severe NDI, 43.8 ± 5.2 weeks, *P* = 0.153). Compared with term-birth controls, preterm infants demonstrated distinct alterations in FC patterns associated with their varying severities of NDI outcomes (Fig. 2A). A clear trend emerged within the preterm cohort: as NDI severity increased from the no/mild group to the moderate or severe group, clustering coefficient (reflecting local network connectivity) progressively increased (Fig. 2B), while global efficiency (reflecting overall network integration) declined (Fig. 2C). Statistical comparisons between the no/mild and moderate/severe groups confirmed these patterns (clustering coefficient: *t* = −4.38, *P* < 0.001; global efficiency: *t* = 3.44, *P* < 0.001). Furthermore, the moderate/severe NDI group exhibited significantly lower global efficiency than the no/mild NDI group, suggesting a shift toward less integrated and more segregated network organization with increasing NDI severity.

**Figure 3:**
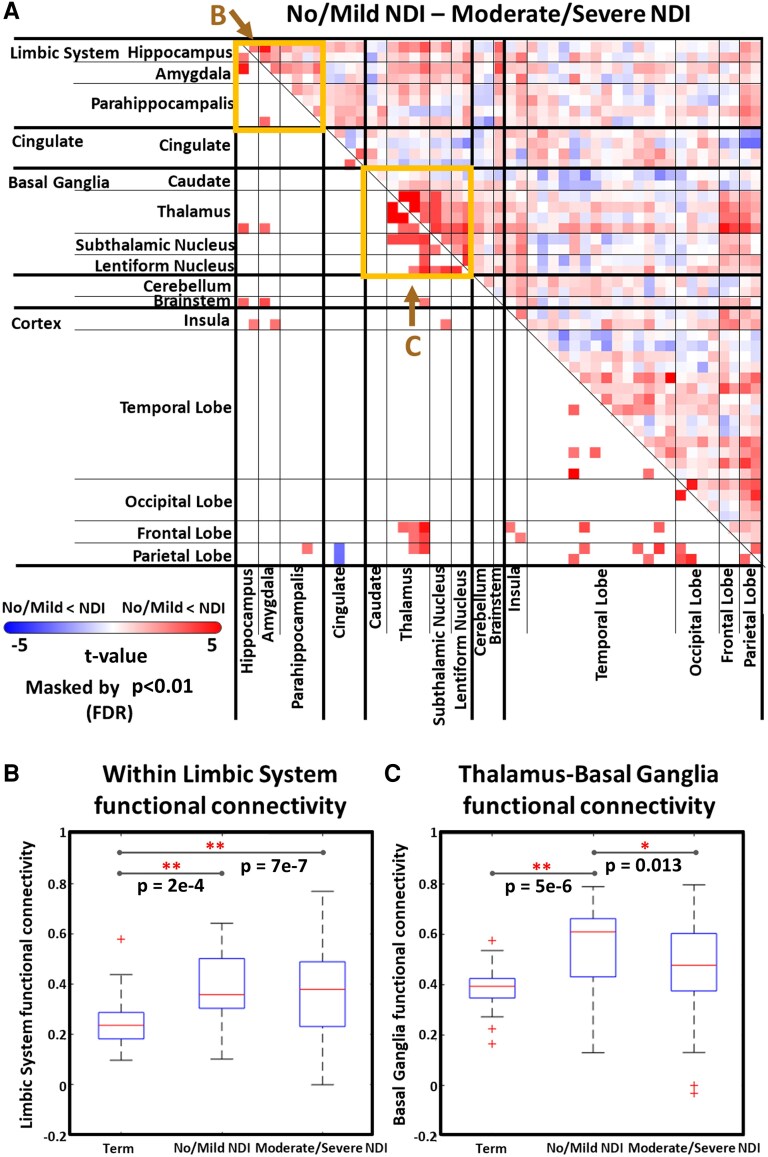
**Differences in FC at term-equivalent age.** (**A**) Comparison between preterm infants with no/mild NDI (*N* = 89) and those with moderate/severe NDI (*N* = 33). Specific comparisons for connectivity within the limbic system (**B**) and between the thalamus and basal ganglia (**C**), with ROI indicated by boxes in the corresponding connectivity matrices (A). All FC matrices were masked at a significance level of *P* < 0.01 with FDR correction. (Significance threshold: *P < 0.05 and **P < 0.01 with FDR correction). FDR, false discovery rate; NDI, neurodevelopmental impairment.

Compared with term-birth controls, the no/mild NDI group showed elevated FC within the limbic system, and between the thalamus and basal ganglia (Supplementary Fig. 1A). However, this effect appeared to be weakened gradually in the moderate NDI (Supplementary Fig. 1B), and especially in the severe NDI group (Supplementary Fig. 1C). In these cases, the increased thalamus-basal ganglia connectivity observed in the no/mild NDI group was significantly higher than in the moderate/severe group, indicating group-level differentiation in this subcortical circuit (Fig. 3A). FC within the limbic system remained elevated in both the no/mild and moderate/severe groups relative to term controls, without a significant difference between the two preterm infant groups with differing levels of NDI (Fig. 3B). In contrast, a thalamus–basal ganglion FC was significantly higher in the no/mild group compared with both term and moderate/severe groups (Fig. 3C). These findings highlight differential connectivity patterns across NDI severity levels within subcortical circuits.

### Brain FC patterns at term-equivalent age associated with cognitive, language and motor performance at age 24 months

**Figure 4:**
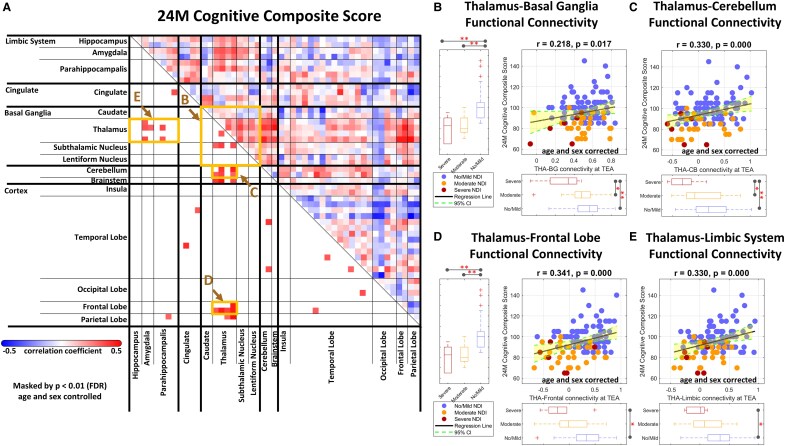
**Association of FC at term-equivalent age with cognitive outcomes at age 24 months.** (**A**) A correlation matrix displaying partial Pearson correlation coefficients between specific connectivity measures and the cognitive composite score in preterm infants (*N* = 122), with ROI for (**B–E**) indicated by boxes. Significant linear regression relationships are shown between cognitive scores and connectivity at (**B**) thalamus–basal ganglia, (**C**) thalamus–cerebellum, (**D**) thalamus–frontal lobe, and (**E**) thalamus–limbic system. Partial correlation analyses and regression analyses were performed using data from all three groups combined, no/mild (*N* = 89), moderate (*N* = 27), and severe NDI group (*N* = 6), and were controlled for postmenstrual age at MRI and sex (significance: *P < 0.05, **P < 0.01 with FDR correction). In the scatterplots, each dot represents an individual participant, with blue indicating the no/mild NDI group, orange indicating the moderate NDI group, and red indicating the severe NDI group. CI, confidence interval; FDR, false discovery rate; NDI, neurodevelopmental impairment; TEA, term-equivalent age; THA**–**BG, thalamus-basal ganglia; THA**–**CB, thalamus-cerebellum.

**Figure 5:**
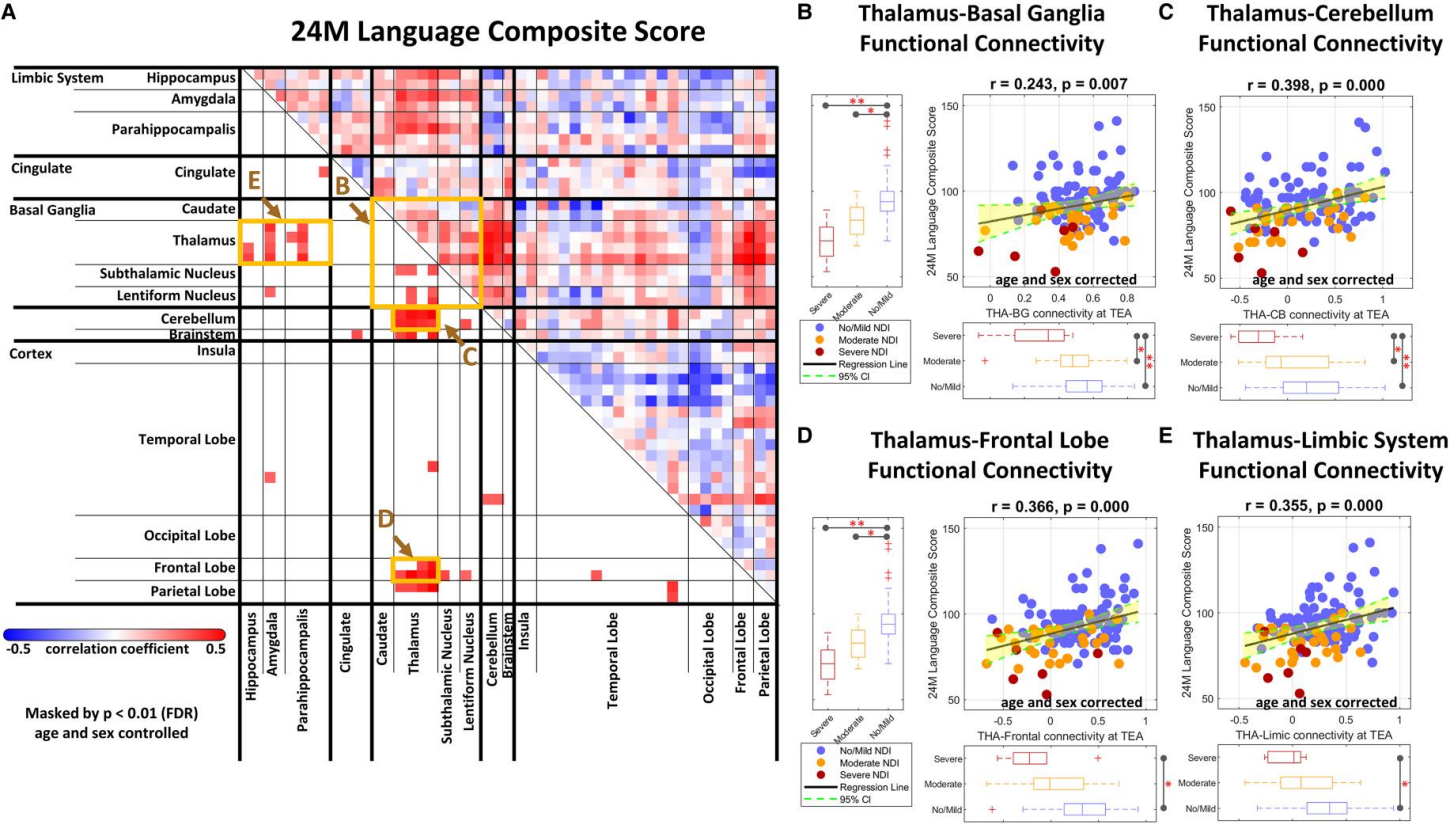
**Association of FC at term-equivalent age with motor outcomes at age 24 months.** (**A**) A correlation matrix displaying partial Pearson correlation coefficients between specific connectivity measures and the motor composite score in preterm infants (*N* = 122), with ROI for (**B–E**) indicated by boxes. Significant linear regression relationships are shown between cognitive scores and connectivity at (**B**) thalamus–basal ganglia, (**C**) thalamus–cerebellum, (**D**) thalamus–frontal lobe, and (**E**) thalamus–limbic system. Partial correlation analyses and regression analyses were performed using data from all three groups combined, no/mild (*N* = 89), moderate (*N* = 27), and severe NDI group (*N* = 6), and were controlled for postmenstrual age at MRI and sex (significance: *P < 0.05, **P < 0.01 with FDR correction). In the scatterplots, each dot represents an individual participant, with blue indicating the no/mild NDI group, orange indicating the moderate NDI group, and red indicating the severe NDI group. CI, confidence interval; FDR, false discovery rate; NDI, neurodevelopmental impairment; TEA, term-equivalent age; THA**–**BG, thalamus-basal ganglia; THA**–**CB, thalamus-cerebellum.

**Figure 6:**
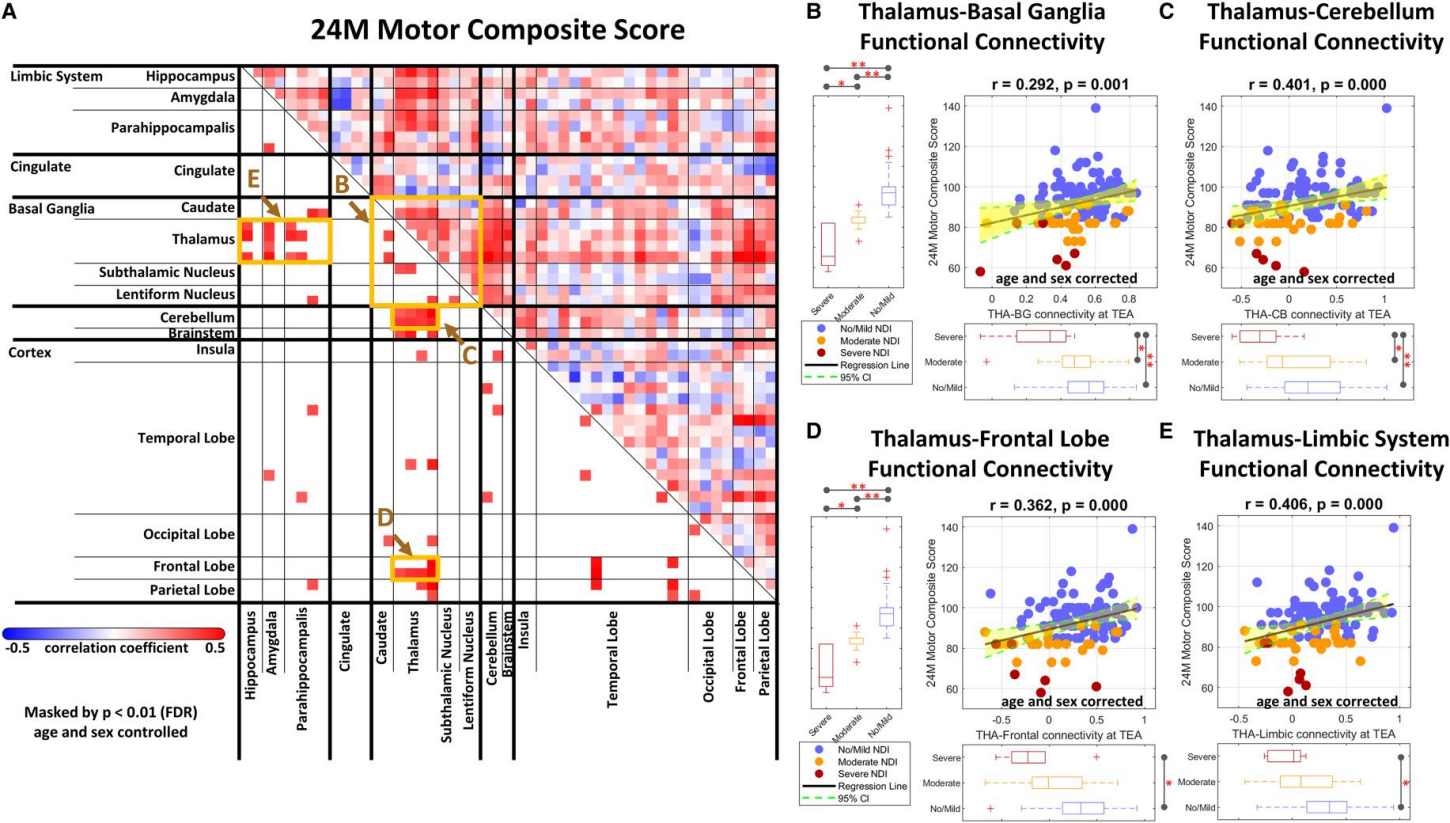
**Association of FC at term-equivalent age with language outcomes at age 24 months.** (**A**) A correlation matrix displaying partial Pearson correlation coefficients between specific connectivity measures and the language composite score in preterm infants (*N* = 122), with ROI for (**B–E**) indicated by boxes. Significant linear regression relationships are shown between cognitive scores and connectivity at (**B**) thalamus–basal ganglia, (**C**) thalamus–cerebellum, (**D**) thalamus–frontal lobe, and (**E**) thalamus–limbic system. Partial correlation analyses and regression analyses were performed using data from all three groups combined, no/mild (*N* = 89), moderate (*N* = 27), and severe NDI group (*N* = 6), and were controlled for postmenstrual age at MRI and sex (significance: *P < 0.05, **P < 0.01 with FDR correction). In the scatterplots, each dot represents an individual participant, with blue indicating the no/mild NDI group, orange indicating the moderate NDI group, and red indicating the severe NDI group. CI, confidence interval; FDR, false discovery rate; NDI, neurodevelopmental impairment; TEA, term-equivalent age; THA**–**BG, thalamus-basal ganglia; THA**–**CB, thalamus-cerebellum.

We further analyzed the respective relationship between the FC patterns at term-equivalent age and the cognitive, language and motor performance at age 24 months of follow-up. Significantly positive correlations were observed between specific FC patterns and the cognitive (Fig. 4A), motor (Fig. 5A) and language (Fig. 6A) outcome scores. Specifically, the cognitive, language, and motor outcome scores were all strongly correlated with FC between the thalamus and basal ganglia (Figs 4B, 5B and 6B), thalamus and cerebellum (Figs 4C, 5C and 6C), thalamus and frontal lobe (Figs 4D, 5D and 6D), as well as thalamus and limbic system (Figs 4E, 5E and 6E). These findings suggest that the thalamo-cortical and thalamo-subcortical pathways play critical roles in neurodevelopment, and may be key indicators of later cognitive, language, and motor outcomes.

**Figure 7:**
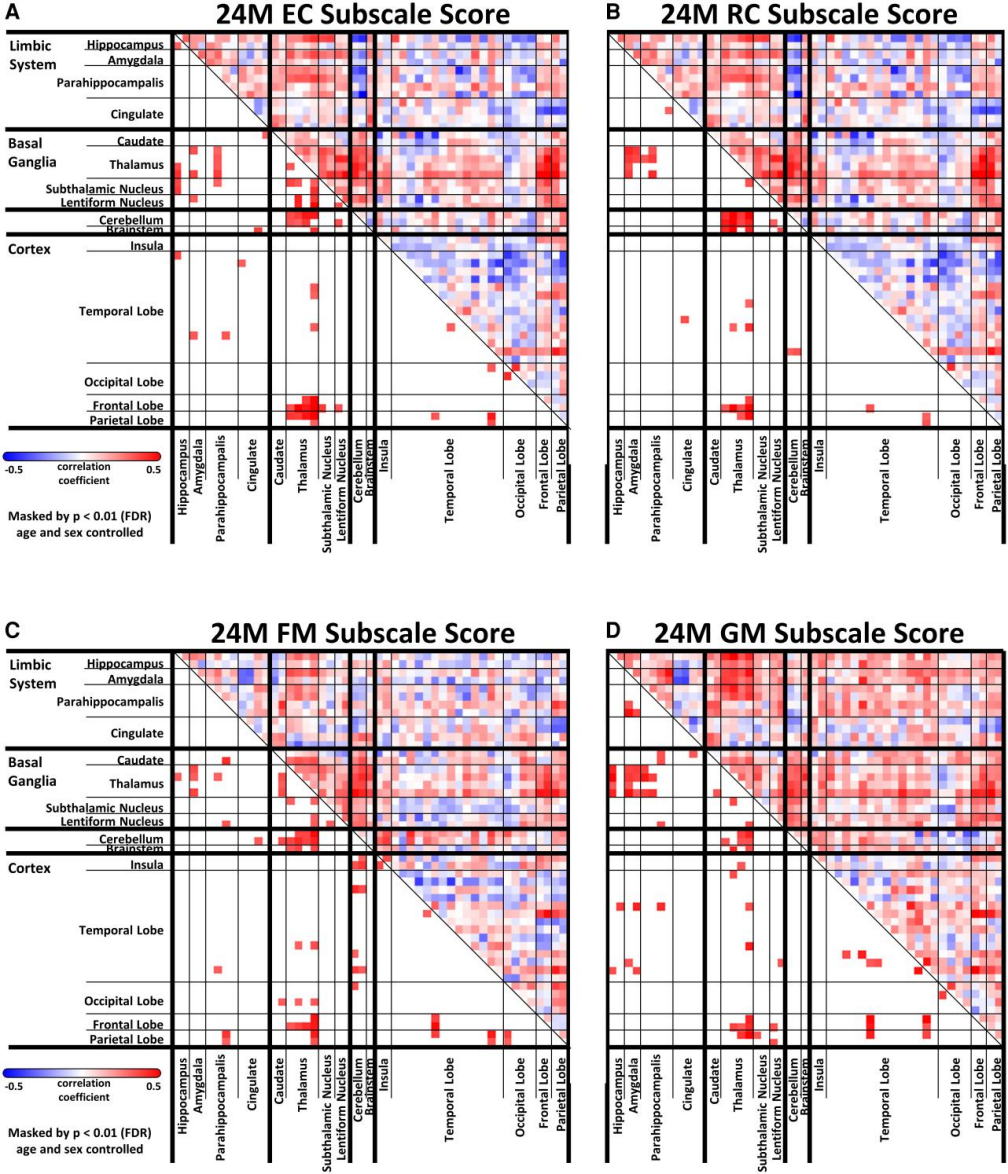
**Connectivity matrices illustrating partial correlations between FC at term-equivalent age and bayley-III subscale scores in the full cohort (*N* = 122).** (**A**) receptive communication (RC), (**B**) expressive communication (EC), (**C**) fine motor (FM), and (**D**) gross motor (GM) domains. Color scale reflects partial Pearson correlation coefficients (r) between connectivity strength and developmental scores, controlling for postmenstrual age at MRI and sex. To assess group-level significance, correlation coefficients were transformed using Fisher’s *Z*-transformation and compared across groups using two-sample *t*-tests with false discovery rate (FDR) correction. EC, expressive communication; FDR, false discovery rate; FM, fine motor; GM, gross motor; NDI, neurodevelopmental impairment; RC, receptive communication.

To further dissect domain-specific associations, we examined correlations between FC at term-equivalent age and individual Bayley-III subscale scores (Fig. 7 and Supplementary Fig. 2). Subscale-specific analyses generally mirrored the patterns observed using composite scores, with significant positive correlations between thalamic connectivity and cognitive, language, and motor functions. Notably, EC scores demonstrated stronger associations with thalamus–basal ganglia connectivity than RC (Fig. 7A, B), suggesting a potentially greater reliance of expressive language on subcortical-motor integration pathways. Similarly, GM scores were more strongly correlated with thalamus–limbic connectivity compared with FM (Fig. 7C, D), potentially reflecting the role of subcortical-limbic circuits in broader motor coordination and postural control. These results support the hypothesis that while thalamocortical and thalamosubcortical pathways contribute broadly to neurodevelopment, distinct circuits may preferentially support specific cognitive or motor subdomains.

**Figure 8:**
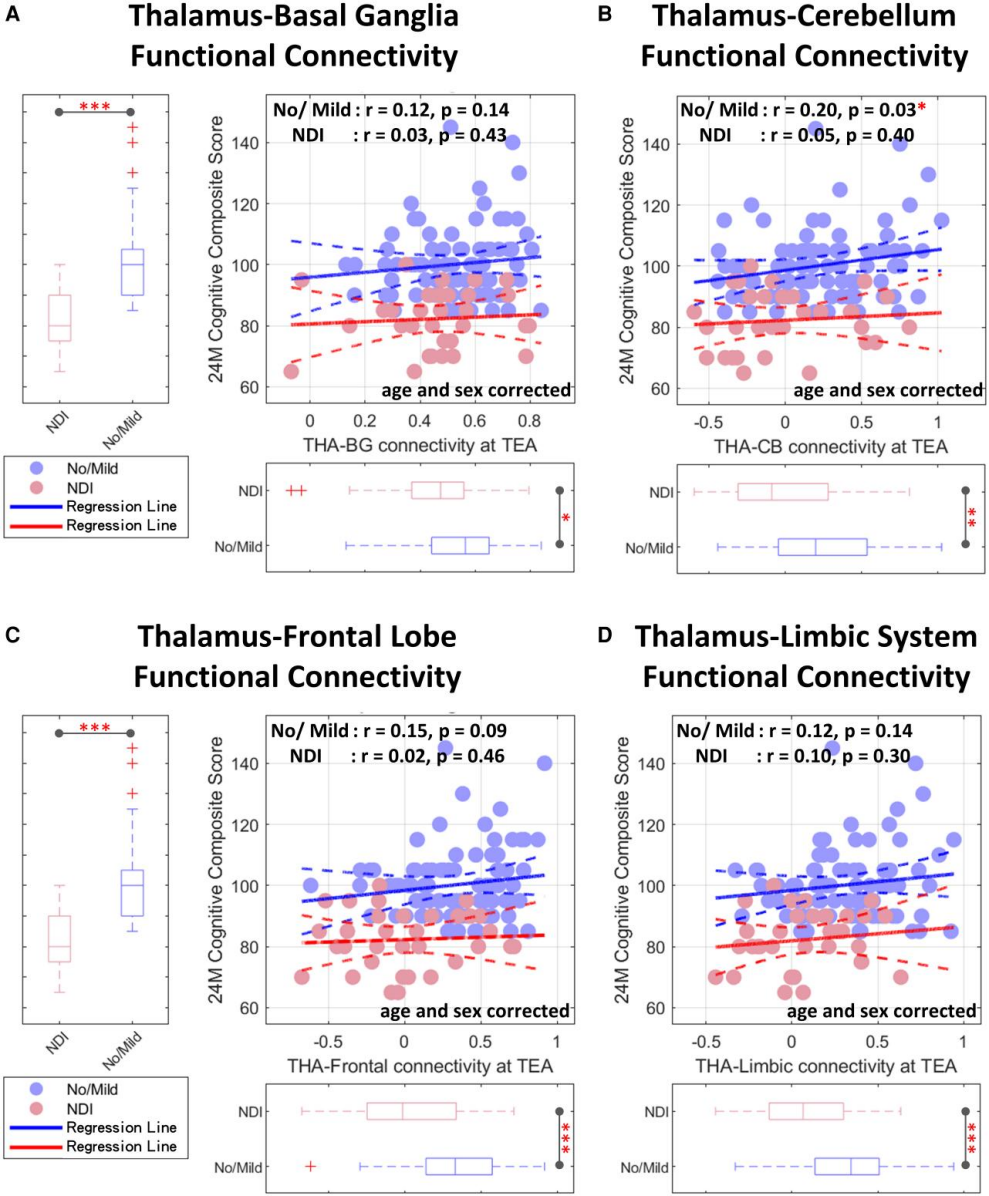
**Group-stratified linear regression plots showing associations between thalamic FC at term-equivalent age and bayley-III composite scores at 24 months.** (**A–D**) Cognitive composite scores plotted against connectivity strength at (**A**) thalamus–basal ganglia, (**B**) thalamus–cerebellum, (**C**) thalamus–frontal lobe, and (**D**) thalamus–limbic system. In the scatterplots, each dot represents an individual participant, with light blue indicating the no/mild NDI group (no/mild; *N* = 89), and pink indicating the moderate/severe NDI group (NDI; *N* = 33). Blue regression lines represent the no/mild NDI group; red lines represent the moderate/severe NDI group. Regression analyses were adjusted for postmenstrual age at MRI and sex. (Significance: *P < 0.05, **P < 0.01 with FDR correction). CI, confidence interval; FDR, false discovery rate; NDI, neurodevelopmental impairment; TEA, term-equivalent age; THA**–**BG, thalamus-basal ganglia; THA**–**CB, thalamus-cerebellum.

To explore whether the observed associations between FC and neurodevelopmental outcomes were driven primarily by between-group differences or also present within each of the different NDI outcome group, we conducted stratified correlation analyses within the no/mild and moderate/severe groups separately (Fig. 8 and Supplementary Fig. 3). For each group, we assessed the relationships between connectivity in key thalamocortical and thalamosubcortical pathways and cognitive, language, and motor scores. While the overall patterns of association remained directionally consistent, the majority of within-group correlations did not reach statistical significance after correction for multiple comparisons. For instance, thalamus–basal ganglia connectivity remained positively associated with cognitive scores in both groups but did not reach significance within either subgroup individually. Similar non-significant trends were observed for language and motor domains. This finding suggests that the robust associations observed across the full sample may be largely driven by between-group level differences in both FC and developmental outcomes.

### Individualized prediction of NDI outcomes at age 24 months

**Figure 9:**
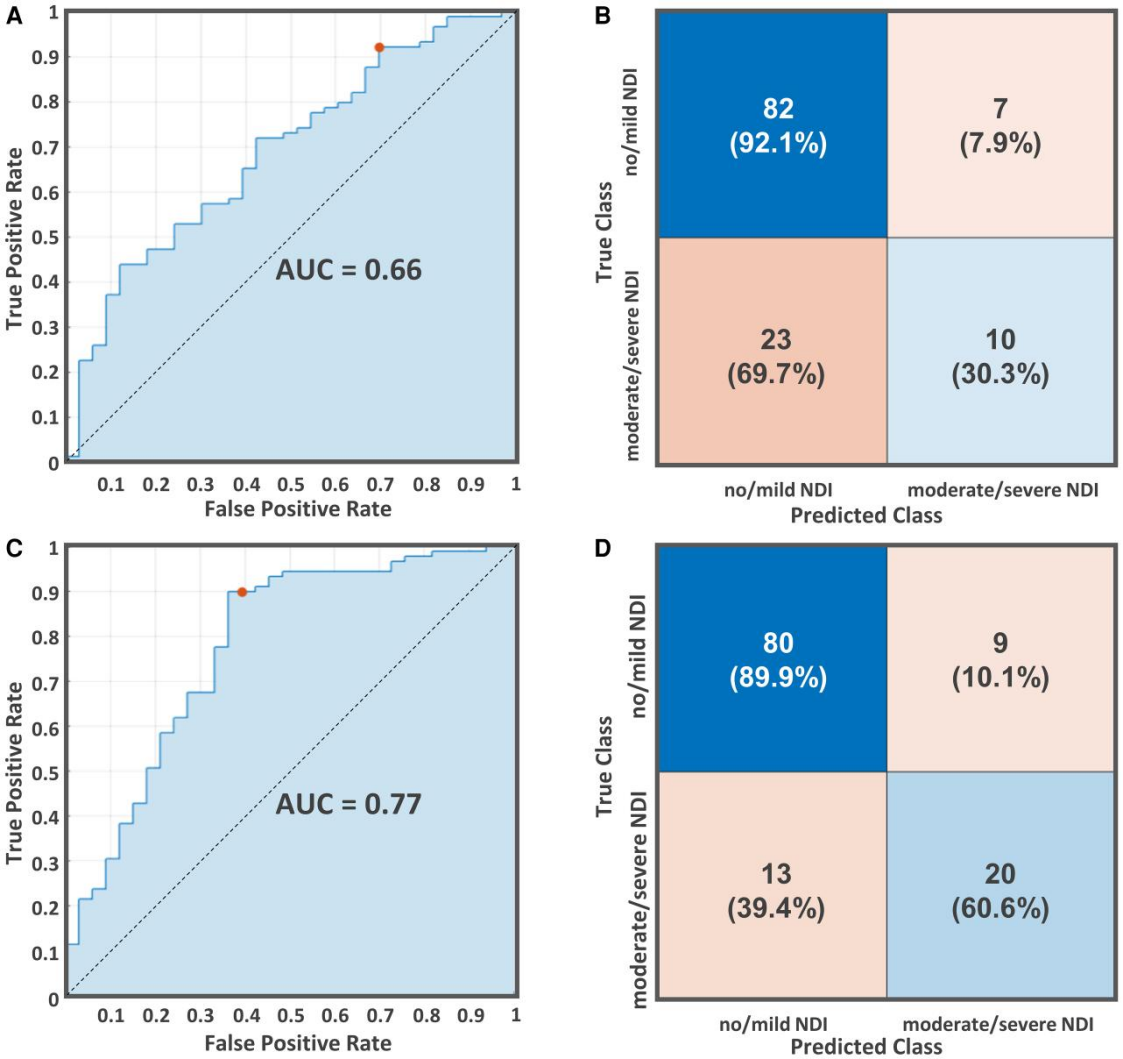
**Predictive models for NDI outcomes at 24 months.** (**A**) Receiver operating characteristic (ROC) curve for a model using the selected neonatal adverse exposure features, including GA at birth, durations of IMV, oxygen therapy, vasopressor use, and total parenteral nutrition, along with postmenstrual age and sex to differentiate no/mild NDI (*N* = 89) from moderate/severe NDI (*N* = 33). (**B**) Corresponding confusion matrix for this model. (**C**) ROC curve for an enhanced model incorporating FC features with neonatal exposure parameters. (**D**) Confusion matrix summarizing the enhanced model's performance. The red dots in ROC curves represent the optimal operating points, corresponding to the thresholds that provide the best trade-off between sensitivity and specificity. AUC, area under curve.

For the prediction of NDI outcomes, we first used adverse neonatal exposures during admission and confounding factors (postmenstrual age at MRI scan and sex) to predict NDI outcomes (Supplementary Table 1). The Lasso method identified important neonatal exposures predictors, including GA at birth, durations of IMV, oxygen therapy, vasopressor use, and total parenteral nutrition, along with postmenstrual age at MRI scan and sex. Among all tested models, the SVM classifier showed the best performance, achieving an accuracy of 75.4% and an ROC-AUC of 0.66 (Fig. 9A, B).

Next, we examined whether incorporating rsfMRI biomarkers—including graph theory metrics (clustering coefficient and global efficiency) and FC patterns—could improve the predictive power for NDI. The Lasso method identified important predictors: GA, sex, durations of IMV, four key FC patterns (thalamus-basal ganglia, thalamus-frontal lobe, thalamus-cerebellum, and thalamus-limbic system), and postmenstrual age at MRI scan. Incorporating these features, the K-Nearest Neighbor classifier yielded the best performance, with an accuracy of 82.0% and an ROC-AUC of 0.77 (Fig. 9C, D and Supplementary Table 1).

## Discussion

Severe neonatal brain injury can disrupt critical brain connectivity, leading to NDI in preterm infants. However, even without severe neonatal brain injury, preterm infants remain at high risk of NDI, and it is unclear whether connectivity alterations at term-equivalent age reflect the heterogeneity of NDI outcomes. Our study revealed that infants who later developed no/mild, moderate, or severe NDI already showed distinct alterations in hub connectivity at term-equivalent age. Four FC patterns involving the thalamus (with basal ganglia, cerebellum, frontal lobe, and limbic system) were linked to cognition, language, and motor outcomes, and also improved predictive accuracy for NDI when combined with neonatal exposures. These findings highlight rsfMRI-detected FC dysmaturation at term-equivalent age as a potential biomarker for early identification of infants at high risk of NDI, even in the absence of severe neonatal brain injury.

### FC as early biomarkers for neurodevelopmental performance

Advances in neuroimaging have enabled clinicians to investigate whether alterations in brain connectivity development commonly identified at term-equivalent age underlie their risk of NDI, including language, motor, and cognitive deficits.^3,40^ Previous studies have demonstrated altered brain connectomes in preterm infants at term-equivalent age compared with that in full-term controls, emphasizing short-range functional connections within networks across various brain regions and identifying reduced global integration in preterm infants.^41^ In this study on infants without severe neonatal brain injury, whole-brain FC was assessed, and graph theory metrics were applied to characterize the topological organization of the developing brain network by term-equivalent age that associated with different NDI outcomes. Compared with term-birth controls, preterm infants who later developed different severity of NDI exhibited significant alterations in connectivity patterns, characterized by decreasing global efficiency and increasing clustering coefficient, indicating a shift toward more localized but less integrated brain networks at term-equivalent age in infants with worsening NDI outcomes at follow-up. Additionally, we observed elevated FC within the limbic system and thalamus-basal ganglia circuits in infants with no/mild NDI outcome. This heightened connectivity likely represents a compensatory mechanism,^42^ wherein the brain enhances specific network pathways involved in memory and sensorimotor integration to counterbalance broader connectivity dysmaturation. However, such compensatory reorganization appeared reduced or absent in preterm infants with moderate to severe NDI severity, suggesting a failure of adaptive network development in these cases.

In the majority of preterm infants, structural MRI does not predict motor or cognitive outcomes accurately, and many infants without apparent brain lesions later develop motor and cognitive deficits.^43^ The thalamo-cortical FC are necessary for normal brain function,^44^ which develop during late fetal life and are vulnerable to neonatal adversity exposures.^45,46^ One study had showed that altered cortico-thalamic FC at term-equivalent age was associated with increased difficulties with motor and cognitive outcomes at 2 years old in infants without severe neonatal brain injury.^43^ In contrast, another study found that in infants with severe neonatal brain injury, the FC in the interhemispheric motor cortex was positively related to GM outcomes. The thalamo-cortical connectivity was not related to motor scores. In contrast, in infants without severe neonatal brain injury, there was no relationship between FC and motor outcome.^47^ Our research further highlighted the important role of the thalamo-cortical and thalamo-subcortical pathways determined at term-equivalent age in mediating NDI outcomes in infants without severe neonatal brain injury. We also found the thalamo-cortical (frontal lobe, limbic system), thalamo-basal ganglia, and thalamo-cerebellum pathways played critical roles in later cognition, language, and motor function, highlighting the potential contribution to different aspects of neurodevelopmental performance outcomes. These findings suggest that FC dysmaturation at term-equivalent age, possibly reflecting adverse exposures in the NICU,^12,14-16^ may contribute to circuit alterations that predispose infants to varying NDI outcomes.

While the overall pattern of associations was generally consistent with those observed using composite scores, we found subtle domain-specific differences. For example, thalamus–basal ganglia connectivity was more strongly associated with EC than RC, suggesting a stronger link between subcortical circuits and expressive language. Conversely, GM scores showed a stronger correlation with thalamus–limbic connectivity compared with FM, implying that GM development may depend more heavily on subcortical-limbic integration. These findings provide a more nuanced understanding of how early brain connectivity may differentially support specific neurodevelopmental functions.

Although the overall FC–outcome correlations were significant across the full cohort, these associations were generally attenuated and not statistically significant within each group individually. It is possible that the relationship between early brain connectivity and later neurodevelopment may not be strictly linear within each severity group, but rather reflect threshold effects or non-linear trajectories of brain development. Additionally, the limited sample size—particularly in the moderate/severe group—may have reduced the power to detect significant correlations within groups. Future studies with larger samples are needed to clarify whether the relationship between FC and outcome persists within more narrowly defined clinical strata.

### Feature factors predicting NDI outcomes among infants without severe neonatal brain injury

Recently, a systematic review of the machine learning studies in predicting neurodevelopmental outcomes of premature infants, with most studies from brain structure images, suggests promising initial works have been conducted, but many challenges remain.^48^ No machine learning studies have used the resting state FC to predict neurodevelopment outcome. Only one study took rsMRI data from 50 very preterm-born infants without severe neonatal brain injury and 50 term-born control infants to predict brain maturity patterns at term-equivalent age using machine-learning algorithms.^49^ Our findings suggest that thalamus-basal ganglia connectivity, thalamus-frontal lobe connectivity, thalamus-cerebellum connectivity, thalamus-limbic system connectivity at term-equivalent age, and the postmenstrual age at MRI examinations may serve as predictive features for NDI outcomes.

Previous studies have highlighted the importance of thalamus-basal ganglia connectivity in motor control and cognitive processing, emphasizing its pivotal role in early brain development.^50,51^ Consistent with this, we found that dysmaturation in thalamus-basal ganglia pathways were significantly associated with motor and cognitive deficits at follow-up. Additionally, other research has demonstrated the critical role of thalamus-frontal lobe connectivity, particularly in goal-directed behaviour and cognitive functions.^52^ Our study observed similar patterns, with impaired thalamo-cortical connectivity correlating with poorer cognitive outcomes, reinforcing the notion that early dysmaturation in this circuitry may contribute to later NDI. The interconnectivity between the thalamus and cerebellum further underlined the thalamus’s role as a central relay for motor and cognitive functions.^51,53^ In our study, stronger thalamus-cerebellum connectivity was associated with better neurodevelopmental outcomes. Furthermore, the Papez circuit, a pathway involved in memory and emotion regulation, including both the limbic system and the thalamus,^54,55^ supported our observation that impaired thalamus-limbic connectivity was linked to poorer cognitive outcomes in preterm infants. Previous research also has emphasized that early imaging can offer predictive insights into language comprehension and other cognitive skills,^35^ which aligned with our findings that specific connectivity patterns at term-equivalent age are predictive of outcomes at follow-up.

Although numerous potential confounding factors could not be fully controlled, our model achieved an accuracy of 82.0% and an ROC-AUC of 0.77 by using clinical and FC features. These findings demonstrated the added value of integrating four FC patterns, the thalamo-cortical (frontal lobe, limbic system), thalamo-basal ganglia and thalamo-cerebellum pathways, into the predictive models for improving the identification of infants at risk for moderate/severe NDI outcomes.

### Limitations

There are limitations in this study. First, the sample size, although adequate for initial statistical and modeling analyses, may limit the generalizability of our findings to broader populations of preterm infants without severe neonatal brain injury. A larger, multi-center neuroimaging cohort would enhance statistical power and enable more granular machine learning modeling and subgroup analyses.

Second, although we employed LOOCV to rigorously estimate model performance and reduce the risk of overfitting, the absence of an independent external validation dataset remains a limitation. LOOCV is a widely accepted strategy for small-sample predictive modeling, but future studies should aim to test the generalizability of FC-based predictors in separate, prospectively collected cohorts.

Third, the study relies on a single time point of rsfMRI at term-equivalent age, which—while informative for early FC characterization—cannot fully capture the dynamic maturation of functional brain networks across infancy. Longitudinal neuroimaging across early developmental stages would offer deeper insight into the evolving relationship between brain connectivity and neurodevelopmental outcomes.

Fourth, the term-born control data were acquired using a different scanner and protocol from those used for the preterm cohort. Although these controls were included solely for qualitative reference and were not subjected to direct statistical comparison or predictive modeling, the possibility of scanner-related effects on observed trends cannot be completely excluded. To mitigate this concern, we resampled the dHCP rsfMRI data to match the repetition time and scan duration of our institutional dataset, and processed all data using a uniform preprocessing pipeline.^30,31^ Nevertheless, we acknowledge that residual acquisition differences may persist. Future multi-site or multi-cohort studies should consider applying formal harmonization methods such as ComBat^56^ or neuroComBat^57^ to control for scanner-related confounds.

In conclusion, our study emphasizes the strong predictive value of characteristic FC patterns detected at term-equivalent age, enabling early identification of NDI risk in preterm infants without severe neonatal brain injury. Despite the aforementioned limitations, these FC biomarkers hold promise for guiding targeted early interventions to improve neurodevelopmental outcomes.

## Supplementary Material

fcaf476_Supplementary_Data

## Data Availability

Open-source software and datasets are available from the resources as cited, or from the authors upon request. The custom code used for data analysis in this study has been uploaded to a public GitHub repository (available at: https://github.com/YiTienLi/Preterm_infant_study).
