# Supplementary material for: Functional connectivity as a prognostic biomarker for neurodevelopmental outcomes in preterm infants without severe brain injury
Source: Brain Commun. 2025 Dec 16;7(6):fcaf476. doi: 10.1093/braincomms/fcaf476 (PMC12704428; doi:10.1093/braincomms/fcaf476)
Supplement: fcaf476_Supplementary_Data [file fcaf476_supplementary_data.docx]

**Supplementary Materials**

**
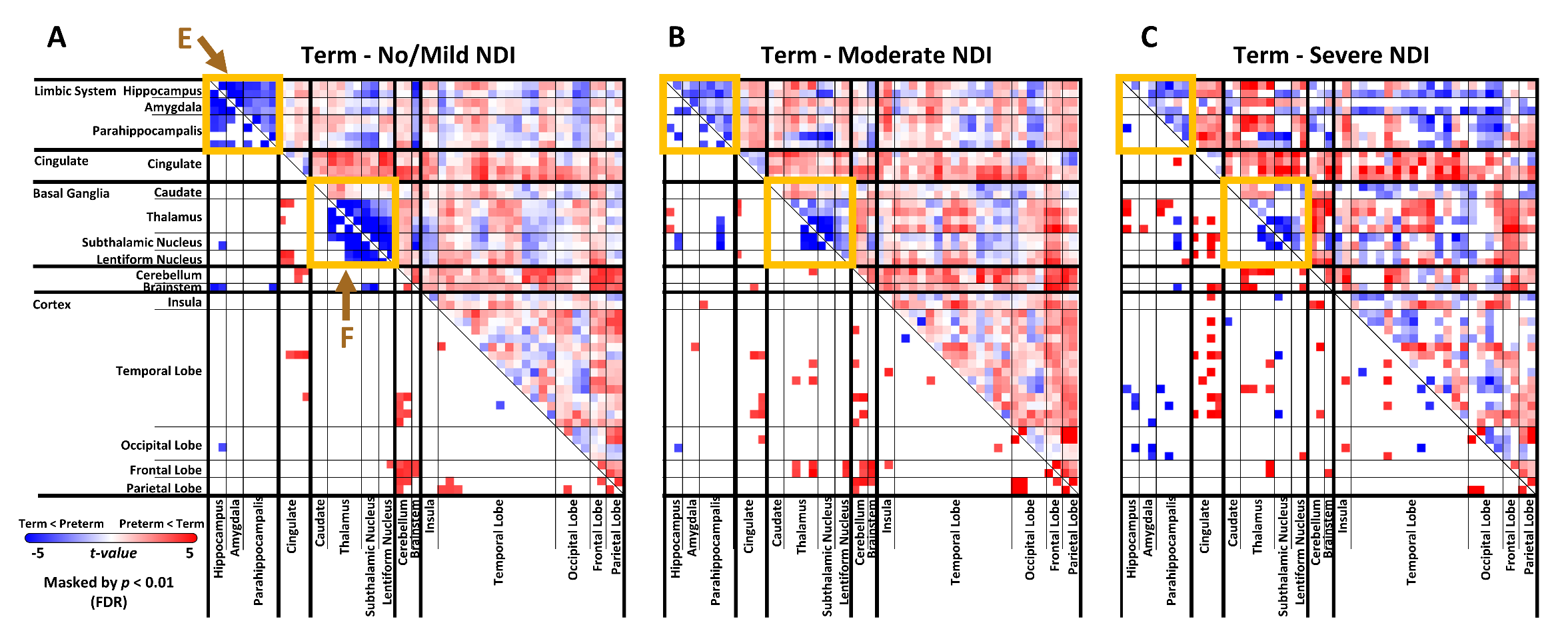
**

**Supplementary Figure 1. Functional Connectivity Matrices at Term-Equivalent Age.** Functional connectivity matrices comparing term-born controls (N = 40) with preterm infants with (A) no/mild NDI (N = 89), (B) moderate NDI (N = 27), and (C) severe NDI (N = 6). All functional connectivity matrices were masked at a significance level of *p* < 0.01 with false discovery rate (FDR) correction. Abbreviations: NDI = neurodevelopmental impairment; FDR = false discovery rate.

**
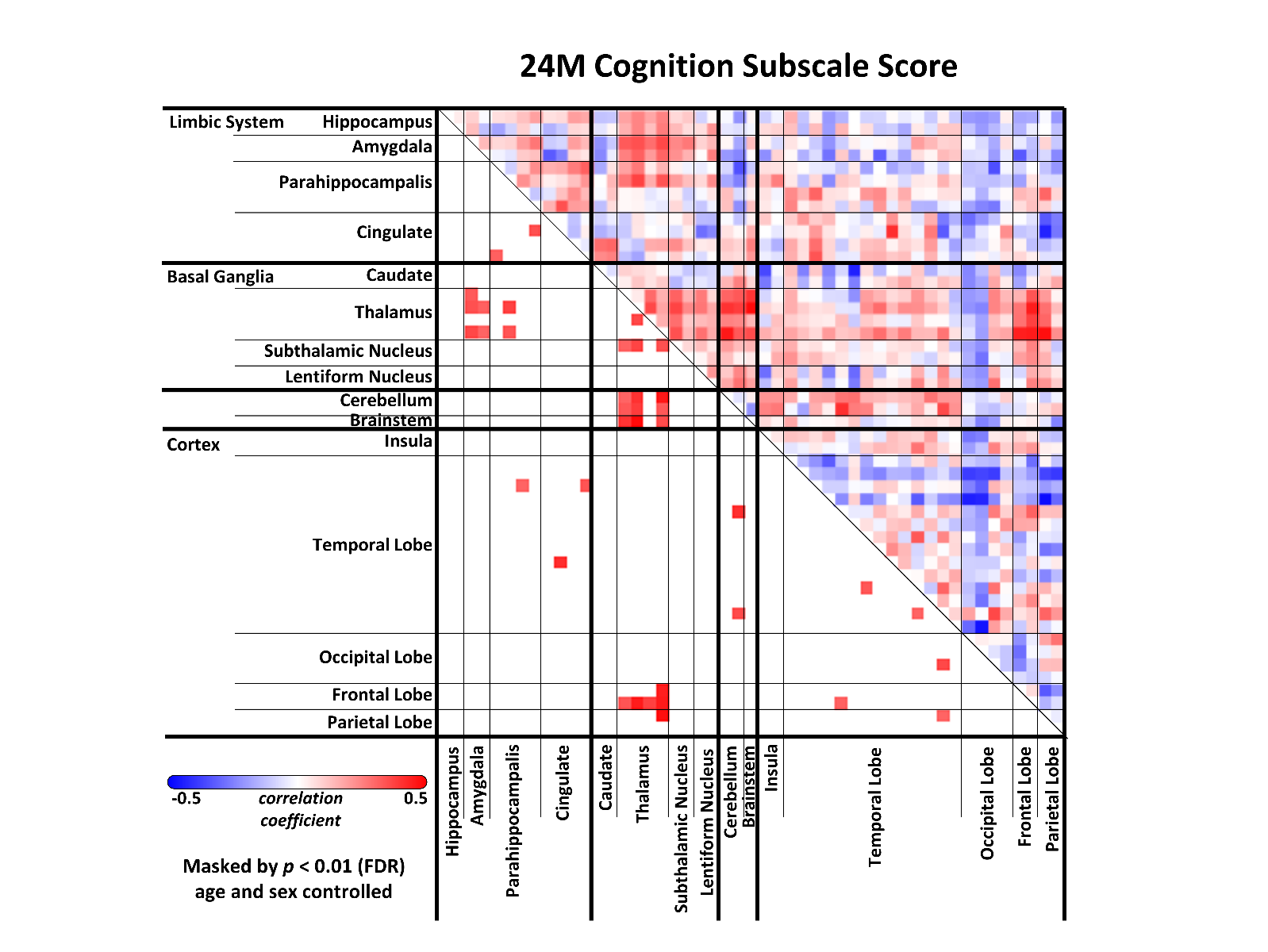
**

**Supplementary Figure 2. Connectivity matrices illustrating partial correlations between functional connectivity at term-equivalent age and Bayley-III cognition subscale scores in the full cohort (N = 122).** (Analyses controlled for postmenstrual age at MRI and sex. Color scale reflects Pearson correlation coefficients. (significance: *p < 0.05, **p < 0.01 with false discovery rate correction). Abbreviations: NDI = neurodevelopmental impairment; FDR = false discovery rate.

**
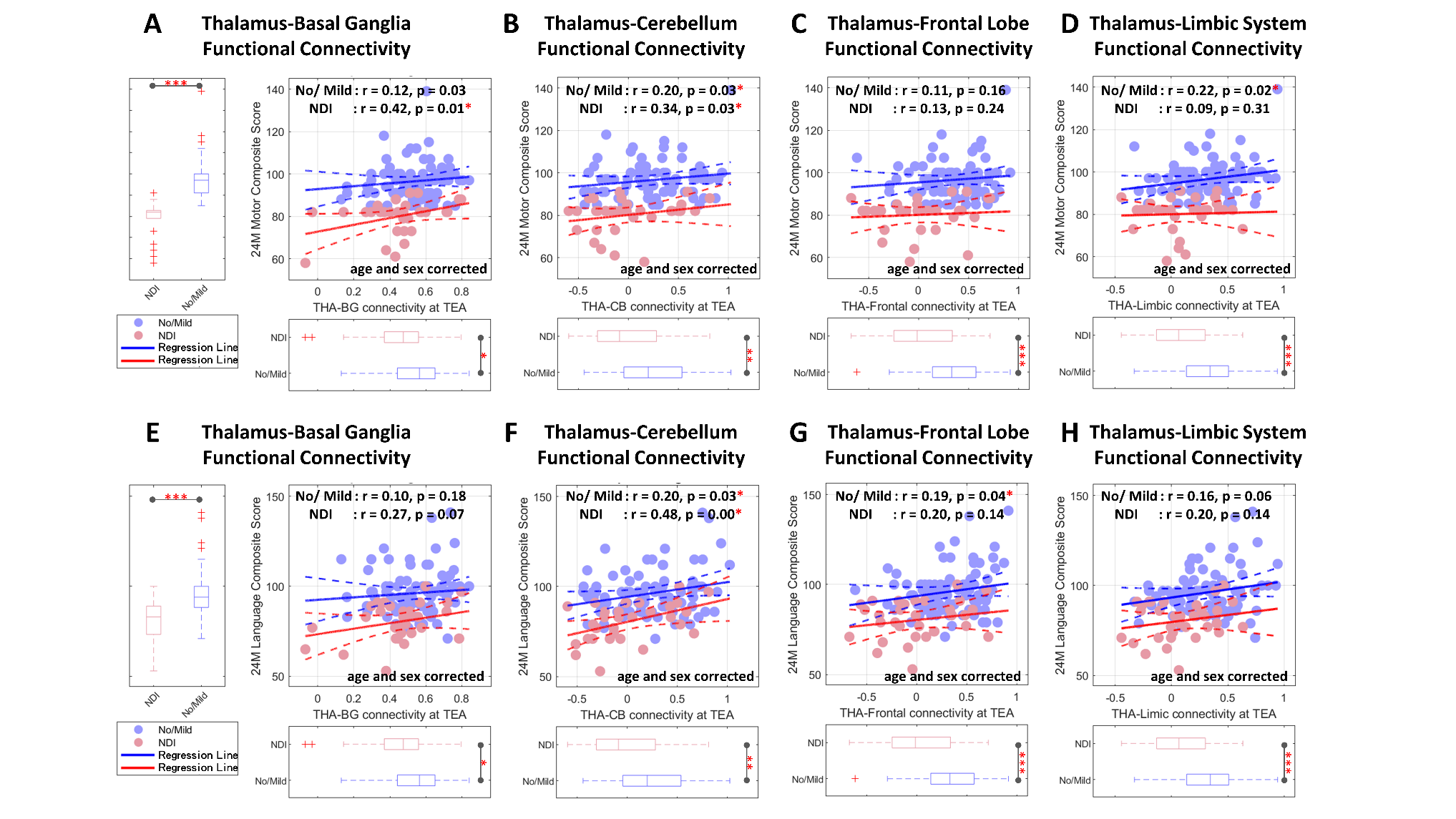
**

**Supplementary Figure 3. Group-Stratified Associations Between Thalamic Connectivity at Term-Equivalent Age and Bayley-III Motor and Language Scores at 24 Months.** (A–D) Motor composite scores plotted against connectivity strength at (A) thalamus–basal ganglia, (B) thalamus–cerebellum, (C) thalamus–frontal lobe, and (D) thalamus–limbic system.

(E–H) Language composite scores plotted against connectivity strength at (E) thalamus–basal ganglia, (F) thalamus–cerebellum, (G) thalamus–frontal lobe, and (H) thalamus–limbic system. Each dot represents an individual participant. Blue regression lines represent the no/mild NDI group (N = 89); red lines represent the moderate/severe NDI group (N = 33). Analyses were adjusted for postmenstrual age at MRI and sex. *p < 0.05, *p < 0.01 with FDR correction.

**Supplementary Table 1. Performance of Prediction Models Using Neonatal Adverse Exposures and Selected Connectivity Features for NDI Outcomes**

| **Model** | **Feature Factors** | **ROC-AUC** | **Accuracy (%)** | **Training Time (s)** |
| --- | --- | --- | --- | --- |
| Decision Tree | neonatal exposures | 0.66 | 64.8 | 30.48 |
| Support Vector Machine | neonatal exposures | 0.69 | 75.4 | 485.86 |
| Naïve Bayes Classifier | neonatal exposures | 0.68 | 68.0 | 97.98 |
| K-Nearest Neighbor Classifier | neonatal exposures | 0.61 | 69.7 | 41.16 |
| Discriminant Analysis | neonatal exposures | 0.72 | 73.0 | 38.84 |
| Ensemble Classifier | neonatal exposures | 0.56 | 72.1 | 352.14 |
| Decision Tree | neonatal exposures + rsfMRI | 0.54 | 78.7 | 33.36 |
| Support Vector Machine | neonatal exposures + rsfMRI | 0.81 | 81.1 | 197.76 |
| Naïve Bayes Classifier | eonatal exposures + rsfMRI | 0.77 | 73.0 | 103.79 |
| K-Nearest Neighbor Classifier | neonatal exposures + rsfMRI | 0.77 | 82.0 | 47.40 |
| Discriminant Analysis | neonatal exposures + rsfMRI | 0.80 | 80.3 | 40.28 |
| Ensemble Classifier | neonatal exposures + rsfMRI | 0.70 | 77.0 | 254.23 |

**Supplementary Table 2**

1 Hippocampus left

2 Hippocampus right

3 Amygdala left

4 Amygdala right

5 Gyri parahippocampalis et ambiens anterior part left GM

6 Gyri parahippocampalis et ambiens anterior part right GM

7 Gyri parahippocampalis et ambiens posterior part right GM

8 Gyri parahippocampalis et ambiens posterior part left GM

9 Cingulate gyrus

10 Cingulate gyrus

11 Cingulate gyrus

12 Cingulate gyrus

13 Caudate nucleus right

14 Caudate nucleus left

15 Thalamus right

16 Thalamus left

17 Thalamus right

18 Thalamus left

19 Subthalamic nucleus right

20 Subthalamic nucleus left

21 Lentiform Nucleus right

22 Lentiform Nucleus left

23 Cerebellum left

24 Cerebellum right

25 Brainstem

26 Insula right GM

27 Insula left GM

28 Anterior temporal lobe

29 Anterior temporal lobe

30 Anterior temporal lobe

31 Anterior temporal lobe

32 Superior temporal gyrus

33 Superior temporal gyrus

34 Medial and inferior temporal gyri anterior part left GM

35 Medial and inferior temporal gyri anterior part right GM

36 Lateral occipitotemporal gyrus

37 Lateral occipitotemporal gyrus

38 Medial and inferior temporal gyri posterior part right GM

39 Medial and inferior temporal gyri posterior part left GM

40 Superior temporal gyrus

41 Superior temporal gyrus

42 Occipital lobe right GM

43 Occipital lobe left GM

44 Lateral occipitotemporal gyrus

45 Lateral occipitotemporal gyrus

46 Frontal lobe right GM

47 Frontal lobe left GM

48 Parietal lobe right GM

49 Parietal lobe left GM
